# Loneliness in Middle-Aged and Older Adults: Effects of Social Environments

**DOI:** 10.3390/bs15010071

**Published:** 2025-01-15

**Authors:** Inna Murtazina, Kristina Krupina, Olga Strizhitskaya

**Affiliations:** Faculty of Psychology, Saint-Petersburg State University, Saint Petersburg 199034, Russia; i.r.myrtazina@spbu.ru (I.M.); st096533@student.spbu.ru (K.K.)

**Keywords:** loneliness, family loneliness, non-family loneliness, social environments, social unconfidence, environmental mastery, sociotropy

## Abstract

Loneliness is a common subjective condition that is associated with distress and negative outcomes for psychosocial functioning and well-being, and it is grounded in destructive or inadequate social functioning. Social interactions are considered one of the key factors determining loneliness, and similarly to social interactions, loneliness can occur in different domains. While a solid body of research is focused on loneliness as a general condition, there are few studies that investigate loneliness from a multidimensional perspective, particularly combining general and domain-specific loneliness. In the present study, we conceptualized loneliness as a complex phenomenon. We focused on the associations between different types of loneliness and the characteristics of social environments. The participants were 140 adults aged 45–73 (58.9% females). The methods involved the Multidimensional Inventory of Loneliness Experience, the Social and Emotional Loneliness Scale (SELSA-S), the “Sociotropy—Self-Sufficiency” Questionnaire, and the assessment of demographic characteristics (age and sex). To test our hypothesis, we applied regression path modeling. The results showed that general loneliness predicted both family and non-family loneliness. We also found that general loneliness increased experiences of social uncertainty, while non-family loneliness decreased positive relations with others. No age effects were found. An effect of sex was found for social uncertainty and positive relations with others.

## 1. Introduction

Loneliness is a common subjective condition that is associated with distress and negative outcomes for psychosocial functioning and well-being, and it is grounded in destructive or inadequate social functioning (Cacioppo & Cacioppo, 2018). It is a widely spread condition that affects people regardless of age, sex, education, religion, or socioeconomic status. Loneliness is a complex phenomenon that can manifest itself in different domains (for example, family and non-family). It can be attributed to a real lack of relationships and isolation, as well as subjective discrepancies between the desired and actual quality of relationships (Luo et al., 2012). Feeling lonely can be described in terms of closeness, intimacy, emotional support, and connectedness. The phenomenon of loneliness has a complicated nature, as it to some extent relates to one’s emotional sphere, but at the same time, it is grounded in the quality and quantity of social interactions that a person has, as well as individual differences. In the literature, there are two commonly used terms that are associated with loneliness. They are emotional loneliness and social isolation. The first corresponds to the quality of relationships, relating to a lack of mutual understanding or support, while the second is associated with the deprivation of social interaction (Wolters et al., 2023). Although loneliness is usually associated with negative outcomes, one of its components—solitude—is believed to also have positive effects. Solitude—or sometimes, positive solitude—is a form of loneliness that reflects an internal need to be alone. Usually, it is described as a temporary state that one needs for self-understanding, self-analysis, and planning (Ost Mor et al., 2020). Sometimes this type of loneliness is also called “dynamic loneliness”.

There is a solid body of research revealing the negative effects of loneliness. It is associated with a variety of significant risks for health and well-being, such as depression (Cacioppo et al., 2006; Prizeman et al., 2023), psychological stress and anxiety (Yanguas et al., 2018), and all-cause premature death (for review, see Holt-Lunstad et al., 2015; Lennartsson et al., 2022). Although there is a solid body of research on loneliness, its mechanisms and factors remain understudied due to the complexity of the phenomenon and the variety of its manifestations. Studies on the risk factors of loneliness show that age and sex have quite complex effects (Barjaková et al., 2023). The results depend on the measures used and the types of loneliness studied. Many demographic factors, such as race, education, and employment, remain understudied and are taken only as control variables.

The analysis of studies from 78 countries revealed that there is no direct association between loneliness and age (Mund et al., 2020). Mund et al. (2020) suggested that loneliness could be predicted not only by social situation but to some extent by personality characteristics. Buecker et al. (2020) argued that extraversion, neuroticism, and agreeableness were somewhat related to experiencing loneliness. These results were replicated by Erevik et al. (2023). The authors also hypothesized that associations between personality traits and loneliness could be contradictory, and to some extent, loneliness could affect the manifestation of personality traits. The authors suggest that loneliness could be seen at different levels of functioning. On the one hand, it is a characteristic that reflects a social situation, but on the other, it could be a trait-like characteristic. In other words, loneliness is activated by social situations, but the intensity of the social situation needed to experience loneliness or the intensity of the very loneliness experience could depend on trait-like loneliness.

Considering loneliness as a trait-like characteristic, we come across the question of to what extent genetics contribute to loneliness. The data in the field are quite controversial. Some authors, particularly within evolutionary theory, suggest that there could be genes, or even networks of genes, contributing significantly to the experience of loneliness (for review, see Goossens et al., 2015). At the same time, other authors suggest that there are no direct effects of genetics on loneliness experiences (for review, see Spithoven et al., 2019). In both cases, the authors agree that further investigations on the genetics of loneliness are needed to make reasonable conclusions. Thus, in our study, we consider loneliness as a life-developed characteristic.

Loneliness is presented in all age populations (Böger & Huxhold, 2018). Nevertheless, no reliable data are available on the dynamics of loneliness across the lifespan. Research has shown that loneliness is more likely to affect social integration, while the reverse path is much weaker (Böger & Huxhold, 2018). One possible explanation is that while loneliness is to some extent related to personality traits, it is also related to one’s social environment, and the characteristics of a social environment can be somewhat situational.

While many authors suggest that loneliness is a complex phenomenon (Luo et al., 2012; Buecker et al., 2020), in most empirical studies, loneliness is investigated from a unidimensional perspective. Some authors suggest that while loneliness is linked to cultural differences like individualism or collectivism (Hartanto et al., 2024), one of the possible understudied variables could be the population composition and particularly the characteristics of a community or neighborhood one lives in (Luhmann et al., 2023). Still, the amount of studies in this field remains scarce. Our study conducted during the pandemic (Strizhitskaya et al., 2021b) showed differences in experiencing loneliness during the pandemic’s isolation. We analyzed family, non-family, and romantic loneliness in middle-aged adults. We identified that only the non-family domain suffered from the pandemic’s isolation, although virtual communication remained. Thus, at the same time, people were not lonely within their family context and were lonely from a non-family perspective. These results somewhat support the idea that there could be generalized, so-called trait-like loneliness and domain-specific loneliness.

Loneliness is closely related to social belonging and affiliation (Hawkley & Cacioppo, 2010). From the evolutionary perspective, the need for social belonging is as important as hunger, thirst, or sex (Baumeister & Leary, 1995; Danvers et al., 2023). Some authors argue that intensive experience of loneliness could be interpreted as a motive for re-affiliation: it signals that one is on the social periphery, and it is necessary to re-engage in social relationships (Spithoven et al., 2019; Qualter et al., 2015).

In socioemotional selectivity theory, Carstensen (Carstensen, 1995) suggests that motives for social interactions—key context for loneliness—change over a lifespan. The older one gets, the more importance is given to the quality of relations with others. If we stick to the idea that loneliness is based on a discrepancy between desired and real interactions, socioemotional selectivity theory adds the role of internal subjective changes in the perceptions of relationships. In line with this theory, the Self-Determination Theory (Ryan & Deci, 2000) suggests that, with age, people tend to become more autonomous, but this autonomy does not mean isolation. More likely, it could be some mixture of self-sufficiency and psychological maturity.

From any perspective, loneliness is grounded in the social situation: its quality, quantity, and availability. Thus, the characteristics of social situations and the ability to deal with them, particularly characteristics related to the specifics of the social environment, could affect the intensity of loneliness. One of the possible phenomena that contribute to the understanding of differences in manifestations of loneliness is sociotropy. Similarly to loneliness, the associations of sociotropy and depression were found in many studies (for review, Robins et al., 1994). A meta-analysis of sex differences in sociotropy (Yang & Girgus, 2019) revealed that women demonstrated higher scores on sociotropy although they highlighted the importance of culture in manifestations of sociotropy. Our previous research (Strizhitskaya & Murtazina, 2024) has shown that sociotropy predicts loneliness (general, family, and non-family), but this effect is moderated by autonomy and personal growth. Sociotropy suggests that the need for social affiliation can be caused by self-characteristics, such as the independence of self-confidence, or by the subjective perception of the social environment, such as social uncertainty.

In the present study, we conceptualized loneliness as a complex phenomenon. We believe that different types of loneliness, such as general undifferentiated loneliness and domain-specific loneliness, can be experienced at the same time, so it is important to investigate different types of loneliness using one model. We supposed that the characteristics of the social environment and its subjective perception can affect the experience of loneliness. So in the present research, we focused on the associations between different types of loneliness and the characteristics of social environment. We also took into account age and sex, as their effect in complex loneliness models is understudied. Since there are no consistent data on the directions of interactions between the characteristics of loneliness and the social environment, we expected that these interactions could go both ways.

## 2. Materials and Methods

### 2.1. Data Collection and Research Design

This study was conducted in Saint Petersburg, Russia, from May to November 2023 within the project “Characteristics of experiencing loneliness and ways of coping with it in adulthood”. The total sample of the project was 356 adults aged 30–73. In a previous study (Strizhitskaya & Murtazina, 2024), we used the same sample but with different age intervals (35–45) and focused on moderation analysis. For the present study, we extracted participants aged 40–73 years old. Participants were middle and older adults (n = 186) aged 40–73 (M = 51.8, SD = 8.12, 56.1% females); 83.6% had university degrees, and 64.6% were married. Participants came from different professional backgrounds: education, medicine, management, accounting, engineering, etc. Four participants were excluded due to missing data. The final sample was 180 middle and older adults (Table 1). In the final sample, only 75 participants overlapped with the previous study (Strizhitskaya & Murtazina, 2024).

Participants were recruited via community and social networks. Participants self-reported severe mental and physical health issues that could affect their participation in the study. All participants signed informed consent forms. The study was anonymous, and no personal data, such as names, phone numbers, or addresses, were collected. No incentives were given to the participants. Questionnaires were distributed in person and via online forms (Google Forms).

Our research questions were as follows:

(1) Are there any age or sex effects on loneliness and environmental characteristics?
(2) How is the undifferentiated overall experience of loneliness associated with domain-specific loneliness?
(3) How are different aspects of loneliness associated with environmental characteristics?

Our hypotheses were as follows:

(a) We did not expect age differences in loneliness as there is no relevant consistent evidence for middle-aged and older adults, but we supposed that there could be differences between men and women since women usually manifested increased sensitivity to social interactions;
(b) We tested two alternative hypotheses about the association between general and domain-specific loneliness: general loneliness predicts domain-specific loneliness or vice versa as there are no consistent data in the literature about the nature of their associations;
(c) In our previous studies, we found that sociotropy was associated with general loneliness and psychological well-being (Strizhitskaya & Murtazina, 2024), so we expected to expand those effects to domain-specific loneliness;
(d) In the present study, we supposed that the static characteristics of the environment, such as social unconfidence and positive relations with others, and dynamic characteristics, such as environmental mastery, could have different associations with loneliness;
(e) Finally, we expected sex differences for the effects found in the study.

### 2.2. Measures

For the purposes of the present study, we used two questionnaires to assess loneliness: (1) The Multidimensional Inventory of Loneliness Experience (Osin & Leontiev, 2013), and (2) The Social and Emotional Loneliness Scale (SELSA-S) for Adults and Older Adults (Russian adaptation: Strizhitskaya et al., 2020).

(1) The Multidimensional Inventory of Loneliness Experience. For the present analysis, we used the subscale general experience of loneliness (8 items, Likert scale 1 to 4, α-Cronbach 0.870) that refers to an overall, generalized sense of loneliness (in the analysis, pictures and figures this variable was labeled “overall loneliness”).

(2) The Social and Emotional Loneliness Scale (SELSA-S) for Adults and Older Adults. For the present study, we used scales “Family loneliness” (7 items, Likert Scale from 1 to 5, α-Cronbach 0.898) and “Non-family loneliness” (6 items, Likert Scale from 1 to 5, α-Cronbach 0.890). Family loneliness refers to parents, children, siblings, and spouses; non-family—to friends and colleagues.

We hypothesized that some characteristics of one’s interaction with social and non-social environments could affect the experience of loneliness.

One of the key mechanisms in understanding loneliness is the sense of belonging (Baumeister & Leary, 1995; Vytniorgu et al., 2023). But this mechanism could be affected by the way one perceives the social environment. Thus, we expected that the perception of the social environment as unconfident and unreliable could affect loneliness as well.

In line with socioemotional selectivity theory (Carstensen, 1995) positive relations with others are considered as one of the important factors of healthy and successful aging. It was shown that positive relationships with others increase with age, specifically in the second half of the lifespan.

Environmental mastery is often considered a component of Ryff’s psychological well-being model (Ryff, 1995). For our study, it was important to include a variable that would describe the way one deals with the environment as an active component of social interaction. While many studies acknowledge the associations between environmental mastery and loneliness (for review, see Ben-Zur, 2018), their directions, and latent mechanisms remain understudied.

Particularly, we were interested in the static and dynamic characteristics of the social environment. Social unconfidence and positive relations with others were considered static characteristics as they reflected the subjective state of the social environment. Environmental mastery was treated as a dynamic characteristic as it corresponds to one’s ability to manage the situation (both social and non-social) and see and use the opportunities and resources of this situation.

Social unconfidence was assessed with the “Sociotropy—Self-Sufficiency” Questionnaire (Russian adaptation: Strizhitskaya et al., 2021a). The questionnaire consisted of 39 items that were scored on a Likert Scale from 1 to 5. For the present study, we used a “Social unconfidence” scale (10 items, α-Cronbach 0.813). We also used a short version of the psychological well-being scale (Russian adaptation: Zhukovskaya & Troshihina, 2011) to study environmental mastery and positive relationships with others; each scale had 6 items scored on a Likert Scale from 1 to 5.

### 2.3. Statistical Analysis

Data were processed using the statistical program Statistical Package for Social Sciences (SPSS) (v20.0, SPSS Inc., Chicago, IL, USA) and regression path analysis using AMOS 20.0. An acceptable sample size for path analysis was calculated based on the formula (P × (P + 1)/2 − df) × 10 (Kline, 2011), where P denotes the number of variables and df denotes the degrees of freedom. For our model, the minimum sample size was 170. Thus, our sample (N = 180) was acceptable for analysis.

## 3. Results

First, we analyzed our data for correlations between the variables. Correlation results and descriptive statistics are presented in Table 2.

The results showed no significant correlations with age, significant correlations between all three variables of loneliness, and selective correlations between social unconfidence, environmental mastery, and positive relations and variables of loneliness. Thus, we identified that all the chosen variables except for age were appropriate for inclusion in a path model.

Second, we analyzed our data for sex differences. The results are presented in Table 3.

We found no sex differences in loneliness variables but significant differences in social unconfidence, environmental mastery, and positive relations with others, so sex was also included in the path model since it could affect our background variables.

In our sample, women reported lower levels of loneliness for all three variables, although these differences were not statistically significant. Women also demonstrated higher levels of environmental mastery and positive relations with others. They also had higher scores for social unconfidence. So, these results were somewhat contradictory.

**Figure 1:**
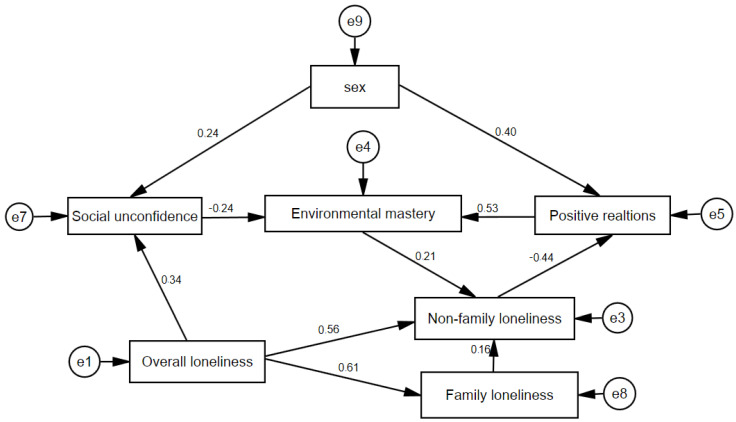
Path model for loneliness and social background variables. Fit indexes for the model: Chi-square = 10.494; df = 11; *p* = 0.487; CFI = 1.000; GFI = 0.984; RMSEA = 0.000; Pclose = 0.793.

Finally, we developed a path model that included overall loneliness that affected family and non-family loneliness. We also included social unconfidence, environmental mastery, and positive relations with others. The results are shown in Figure 1.

Our results proved that overall loneliness predicted family and non-family loneliness. Family loneliness predicted non-family loneliness, but no direct effects were found for social environment characteristics and family loneliness. Overall loneliness increased social unconfidence, despite our expectations of alternative effects. Comparative analysis revealed sex differences for all three social background characteristics. In the path model, only the effects of sex on social unconfidence and positive relationships with others were confirmed.

We found that non-family loneliness affected positive relations with others, and it was affected by environmental mastery. Thus, the more one tries to manage the environment, the more one feels lonely in a non-family social background. At the same time, in accordance with previous research in the field and similarly to the effects of any type of loneliness, non-family loneliness decreased positive relationships with others.

## 4. Discussion

In our study, we focused on the associations between different types of loneliness and the characteristics of the social environment. We grounded in the idea that loneliness could manifest itself in different domains; for example, it could be experienced as a general, overall, undifferentiated feeling of loneliness, or it could be a feeling of loneliness within a specific system (family or non-family). As loneliness is closely related to the social environment and its characteristics, in this study, we focused on their interaction with different experiences of loneliness. We also controlled for age and sex effects.

The modern body of loneliness research is mostly focused on general loneliness without any accents on specific domains, so our results add new data to the field regarding the interactions of different types of loneliness (including well-studied general loneliness) and the different characteristics of social backgrounds.

Our results showed that in the age group of 45–73, there were no age effects on the associations between loneliness variables and variables related to the social environment. Thus, we can assume that these mechanisms could be formed at earlier stages and remain relatively stable throughout adulthood. This result is consistent with the literature (Holt-Lunstad et al., 2015), particularly for middle and older adults.

As Russia implies collectivist and individualist values, our participants could experience some culture-specific effects. On the one hand, family ties are stronger compared to Western countries, but living together for several generations is not common anymore, similarly to many traditional Eastern societies (Hartanto et al., 2024). So, to some extent, we can expect that loneliness in the studied age group could be related to their developmental stage. At this age, we could expect some effects of the “empty nest” syndrome. Still, while adult children do not live with their parents anymore, they maintain close relationships with them. Consistently with this idea, we found that during COVID-19, adults were experiencing more loneliness in non-family environments, particularly older adults (Strizhitskaya et al., 2021b). On the other hand, as adult children leave their parents’ “nest”, they start to make their own “nest”, and parents become involved in grandparenting, which could decrease the possible effects of loneliness.

We did not find direct effects of sex on loneliness, but we found these effects for social environment characteristics. Out of the three effects found in comparative analyses, two effects remained in the path model. Our results suggest that women experience more social unconfidence followed by lower levels of environmental mastery. At the same time, women showed higher estimates for positive relationships with others that supported higher levels of environmental mastery. Thus, we can assume that environmental mastery (which affects non-family loneliness) was affected by two controversial trends, and these trends were reciprocal for men and women. This result, to some extent, is in line with the research on sex differences in sociotropy (Yang & Girgus, 2019). The literature suggests that women overall are expected to have higher scores with respect to sociotropy and related characteristics. Thus, they could be more sensitive to changes in the social environment.

We tested two alternative hypotheses about the associations between general and domain-specific loneliness. We supposed that general loneliness can predict domain-specific loneliness if it reflects the overall feeling of loneliness that is spread to all domains of social interactions. At the same time, domain-specific loneliness could develop into a general feeling of loneliness, and in this case, they would predict general loneliness. To avoid biases related to the development of the questionnaire and to estimate general and domain-specific loneliness, we used instruments developed by different authors. Correlation analyses revealed strong significant associations between all three characteristics of loneliness, but the regression pathway model showed that general loneliness predicts domain-specific loneliness. Alternative pathways were not significant and did not fit the data. This result returned us to the idea that, consistent with Buecker et al. (2020), the general feeling of loneliness could be a trait-like characteristic, and it could make a person more or less prone to experiencing loneliness in general.

The results revealed that family loneliness predicted non-family loneliness. We could hypothesize that family represents the most significant relationship. They develop first during a lifespan and, as many psychodynamic approaches suggest, impact one’s whole life. So, we can suppose that experiences of loneliness within the family increase the chances of having similar feelings outside the family.

We found that general or overall loneliness predicted social unconfidence. This result argues that general loneliness could be interpreted as a trait-like characteristic. It gives some room to speculate that it is not the social environment that makes one feel lonely, but it is one’s loneliness that makes one interpret the social environment as unconfident. This result needs further research but opens an interesting perspective for understanding the nature of loneliness and its possible mechanisms.

One of the interesting results was that environmental mastery, or in other words the ability to manage the situation and resources, predicted non-family loneliness. The alternative pathway was not significant and did not fit the data. One of the possible explanations is that the one who is good at managing situations and operating resources and one who is overall quite capable of one’s own life could feel that all important decisions and actions are those that he(she) does alone, with no assistance or help from others. The most common definition of loneliness states that loneliness is a discrepancy between the desired social interactions (including emotional and psychological support) and actual interactions. So, a possible explanation for this phenomenon would be that a stronger ability with respect to managing a situation and using resources might cause a lack of actual support and thus increase non-family loneliness.

We found no direct pathways between family loneliness and the characteristics of the social environment. At the same time, family loneliness predicted non-family loneliness. We can assume that overall non-family loneliness is more sensitive to characteristics of the social environment. Also, it is possible that the variables in our study provoked association with the social environment in general, rather than with a specific group. It is interesting to suggest that there might be internal separation between the social environment in general and family in particular. In other words, when asked about social interactions, one could exclude family interactions because the family could be considered as more of a kin relationship, which is too important to be considered just social.

Our study has several limitations. First, we used quantitative data to approach possible mechanisms, but qualitative data would allow us to see more individual patterns of loneliness, particularly in domain-specific types of loneliness. Second, there could be some effects caused by the specifics of living in a big city (higher demands and fewer opportunities for intimate social interaction). Third, we did not control for any effects of the “empty nest” or the effects of grandparenting. Finally, the majority of the sample had a university degree, which could affect the results, especially for environmental mastery. This limitation is caused by the high importance that is given to university degrees in Russia, particularly for those aged 45+, and the high overall rates of people with higher education in Russia, particularly in big cities. The limitations of the study open a perspective for future research. They uncover the factors that need a deeper understanding and specific samples for their analysis.

## Figures and Tables

**Table 1:** Demographic characteristics of the sample (N = 180).

Sex		
	Females (%)	56.1
	Males (%)	43.9
Age		
	Range	40–73
	M	51.07
	SD	8.07
Education		
	10 years or less (%)	0.6
	10 to 15 years (%)	16.0
	15 years and more (%)	83.3
Marital status		
	Single (%)	6.1
	Married (%)	63.9
	In a relationship (%)	10.0
	Divorced (%)	13.9
	Widowed (%)	6.1
Occupational status		
	Working (%)	94.1
	Retired (%)	5.9

**Table 2:** Descriptive statistics and correlation analysis for the variables in the study.

	**1**	**2**	**3**	**4**	**5**	**6**
Correlations (r (*p*))						
Age	0.013 (0.858)	−0.057 (0.446)	−0.001 (0.988)	0.153 (0.041)	0.016 (0.832)	−0.032 (0.665)
1. Overall loneliness	1					
2. Family loneliness	0.605 (0.000)	1				
3. Non-family loneliness	0.604 (0.000)	0.449 (0.000)	1			
4. Social unconfidence	0.334 (0.000)	0.269 (0.000)	0.224 (0.003)	1		
5. Environmental mastery	−0.303 (0.000)	−0.281 (0.000)	−0.149 (0.045)	−0.207 (0.005)	1	
6. Positive relations with others	−0.301 (0.000)	−0.291 (0.000)	−0.375 (0.000)	0.057 (0.444)	0.455 (0.000)	1
Descriptive statistics						
M	11.50	10.21	10.94	27.83	10.26	11.08
SD	3.55	4.75	4.22	5.36	1.64	1.92

Note. r—Pearson correlation coefficient; *p*—*p*-value; M—mean; SD—standard deviation.

**Table 3:** Comparative analysis of study variables for females and males (Student’s *t*-test).

	Females (N = 81)		Males (N = 59)		t	*p*	Bootstrap CI (95%)	
	M	SD	M	SD			Low Limit	High Limit
1. Overall loneliness	11.34	3.25	11.71	3.90	0.698	0.486	−0.626	1.438
2. Family loneliness	9.92	4.39	10.57	5.17	0.909	0.364	−0.604	2.064
3. Non-family loneliness	10.76	4.02	11.18	4.48	0.653	0.515	−0.848	1.713
4. Social unconfidence	29.00	5.40	26.34	6.13	−3.086	0.002	−4.245	−0.868
5. Environmental mastery	10.56	1.80	9.86	1.33	−2.913	0.004	−1.168	−0.262
6. Positive relations with others	11.78	1.91	10.19	1.54	−6.035	0.000	−2.106	−1.106

Note. M—mean; SD—standard deviation; *p*—*p*-value; t—t-value for Student’s *t*-test.

## Data Availability

The data presented in this study are available upon reasonable request from the corresponding author.
